# Subchronic Exposure to TCDD, PeCDF, PCB126, and PCB153: Effect on Hepatic Gene Expression

**DOI:** 10.1289/txg.7253

**Published:** 2004-09-22

**Authors:** Chad M. Vezina, Nigel J. Walker, James R. Olson

**Affiliations:** ^1^University of Wisconsin–Madison, School of Pharmacy, Madison, Wisconsin, USA; ^2^National Institute of Environmental Health Sciences, Research Triangle Park, North Carolina, USA; ^3^University at Buffalo, Department of Pharmacology and Toxicology, Buffalo, New York, USA

**Keywords:** AhR, HAH, liver, microarray, PCB, TCDD

## Abstract

We employed DNA microarray to identify unique hepatic gene expression patterns associated with subchronic exposure to 2,3,7,8-tetrachlorodibenzo-*p*-dioxin (TCDD) and other halogenated aromatic hydrocarbons (HAHs). Female Harlan Sprague-Dawley rats were exposed for 13 weeks to toxicologically equivalent doses of four different HAHs based on the toxic equivalency factor of each chemical: TCDD (100 ng/kg/day), 2,3,4,7,8-pentachlorodibenzofuran (PeCDF; 200 ng/kg/day), 3,3′,4,4′,5-pentachlorobiphenyl (PCB126; 1,000 ng/kg/day), or 2,2′,4,4′,5,5′-hexachlorobiphenyl (PCB153; 1,000 μg/kg/day). Global gene expression profiles for each exposure, which account for 8,799 gene probe sets contained on Affymetrix RGU34A GeneChips, were compared by principal components analysis. The aryl hydrocarbon receptor (AhR) ligands TCDD, PeCDF, and PCB126 produced very similar global gene expression profiles that were unique from the nonAhR ligand PCB153, underscoring the extensive impact of AhR activation and/or the resulting hepatic injury on global gene expression in female rat liver. Many genes were co-expressed during the 13-week TCDD, PeCDF, or PCB126 exposures, including classical AhR-regulated genes and some genes not previously characterized as being AhR regulated, such as carcinoembryonic-cell adhesion molecule 4 (*C-CAM4*) and adenylate cyclase-associated protein 2 (*CAP2*). Real-time reverse-transcriptase polymerase chain reaction confirmed the increased expression of these genes in TCDD-, PeCDF-, and PCB126-exposed rats as well as the up- or down-regulation of several other novel dioxin-responsive genes. In summary, DNA microarray successfully identified dioxin-responsive genes expressed after exposure to AhR ligands (TCDD, PeCDF, PCB126) but not after exposure to the non-AhR ligand PCB153. Together, these findings may help to elucidate some of the fundamental features of dioxin toxicity and may further clarify the biologic role of the AhR signaling pathway.

2,3,7,8-Tetrachlorodibenzo-*p*-dioxin (dioxin, TCDD) is a persistent environmental contaminant, a human and rodent carcinogen, and the most potent ligand for the aryl hydrocarbon receptor gene (AhR) (Fingerhut et al. 1991; Gu et al. 2000; Kociba et al. 1978; McGregor et al. 1998). The AhR gene also displays affinity for structurally related xenobiotics, including polychlorinated dibenzo-*p*-dioxins (PCDDs), polychlorinated dibenzofurans (PCDFs), and coplanar polychlorinated biphenyls (PCBs) (Denison et al. 2002). Ligand binding and activation of AhR induces nuclear localization and heterodimerization with the AhR nuclear transporter (ARNT) protein (Whitlock 1999). This activated heterodimer binds to cognate *cis*-acting sequences [dioxin response elements (DREs)], located in the 5′-regulatory region of target genes.

A specific subgroup of genes are activated by an AhR-dependent mechanism during dioxin exposure, including (but not limited to) cytochrome P450 (*CYP*)1A1 *CYP1A1*, *CYP1A2*, *CYP1B1*, aldehyde dehydrogenase (*ADH*), NADPH-quinone-oxidoreductase (*NQO1*), glutathione *S*-transferase (*GST*) Ya (*GSTA1*), and UDP-glucuronosyltransferase 1A1 (*UGT1A1*) (Manjunath and Dufresne 1988; Mimura and Fujii-Kuriyama 2003; Schrenk 1998; Sutter and Greenlee 1992). AhR-dependent transcription is required for dioxin toxicity (Bunger et al. 2003), but it is unclear how activation of AhR-dependent genes produces the multiplicity of toxic responses characteristic of dioxin exposure.

As an attempt to characterize AhR-dependent genes and signaling pathways responsible for subchronic dioxin toxicity, the present study evaluated differential hepatic gene expression in female Harlan Sprague-Dawley (SD) rats exposed subchronically (13 weeks) to toxicologically equivalent doses of the AhR ligands TCDD (100 ng/kg/day), 2,3,4,7,8-pentachloro-dibenzofuran (PeCDF; 200 ng/kg/day), 3,3′,4,4′,5-pentachlorobiphenyl (PCB126; 1,000 ng/kg/day), or the non-AhR ligand 2, 2′, 4, 4′, 5, 5′-hexachlorobiphenyl (PCB153; 1,000 μg/kg/day). This gene expression study was performed in conjunction with a cancer bioassay conducted by the National Toxicology Program (NTP), which included interim sacrifices (13, 30, and 52 weeks) to investigate tissue dosimetry, histopathology, and other biochemical and molecular responses throughout the 2-year study.

Subchronic TCDD exposure is associated with numerous toxic responses, and many of these responses may be AhR dependent. Kociba et al. (1976) showed previously that SD rats exposed to high levels of TCDD (1 μg/kg/day) for 13 weeks were subject to mortality, chloracne, thymic atrophy, and a “wasting syndrome” characterized by rapid weight loss and fat redistribution. Antioxidant enzyme expression was enhanced in rats exposed to lower doses of TCDD for 13 weeks (10–46 ng/kg/day; Hassoun et al. 2003), and the hepatotoxic biomarkers serum bilirubin and alkaline phosphatase were elevated in rats exposed to intermediate doses for the same exposure period (100 ng/kg/day TCDD; Kociba et al. 1976). Rats exposed to TCDD for 13 weeks (100 ng/kg/day) also developed cachexia, hepatic hypertrophy, and altered hepatic foci (Kociba et al. 1976), whereas chronic exposure (104 weeks) at this dose resulted in porphyria and cancer of the liver, lung, and oral mucosa (Kociba et al. 1978; NTP 2004a). Although AhR activation likely contributes to the many toxicologic effects produced by subchronic and chronic TCDD exposures, little is known about AhR and non-AhR signaling mechanisms mediating these effects.

The TCDD, PeCDF, and PCB126 exposure doses used in this study were carcinogenic to female SD rats, but tumors and other hepatotoxic effects were not evident until several months after the 13-week interim sacrifice (NTP 2004a, 2004b, 2004c). Thus, evaluation of differential gene expression patterns after 13-week exposures to subchronic halogenated aromatic hydrocarbons (HAHs) may yield important clues about the mechanisms by which these chemicals produce their chronic toxicologic effects, including cancer. Although there have been several attempts to evaluate TCDD-dependent gene expression *in vitro* and *in vivo* (Fisher et al. 2004; Frueh et al. 2001; Martinez et al. 2002; Puga et al. 2000), to our knowledge, this is the first of such to characterize gene expression during long-term, subchronic exposure to carcinogenic doses of TCDD and other dioxin-like chemicals.

## Materials and Methods

### Sample Procurement

Tissues for this study were provided by the NTP (NTP 2004a, 2004b, 2004c) as part of a 2-year bioassay for relative carcinogenic potencies of dioxin-like chemicals. Female Harlan SD rats were exposed 5 days a week by oral gavage to toxicologically equivalent doses of TCDD [toxic equivalency factor (TEF) = 1.0; 3, 10, 22, 46, 100 ng/kg/day], PeCDF (TEF = 0.5; 6, 20, 44, 92, 200 ng/kg/day), PCB126 (TEF = 0.1; 10, 30, 100, 175, 300, 550, 1,000 ng/kg/day), PCB153 (TEF = 0, N/A; 10, 100, 300, 1,000 μg/kg/day), or corn oil:acetone (99:1; vehicle control). Toxicologic dose equivalence was based on the current World Health Organization TEF recommendations (Van den Berg et al. 1998). Subgroups of rats were sacrificed at 14, 31, and 53 weeks (corresponding to 13, 30, or 52 weeks of exposure), and target organs were removed, flash frozen in liquid nitrogen, and stored at −70°C for mechanistic studies.

### RNA Isolation and Hybridization

The present study used liver from female rats exposed to vehicle control or the highest dose of each compound for 13 weeks to ensure that hepatic gene expression was evaluated in the context of carcinogenic exposure doses for TCDD, PeCDF, and PCB126. Frozen hepatic tissue was disrupted by homogenization with a rotor stator homogenizer, and total RNA was isolated with Qiagen RNeasy columns Qiagen Inc., Valencia, CA). There were a total of six rats in each exposure group. Three pools of RNA were created from each exposure group (*n* = 2 rats per pool), similar to the experimental design of Yechoor et al. (2002). Pooled total RNA was further purified using the Qiagen poly(A) RNA isolation kit. RNA integrity was assessed by the Agilent Bioanalyzer 2100 (Agilent Technologies, Palo Alto, CA). This study employed high-quality RNA that displayed two distinct, sharp peaks and a 28S/18S ribosomal RNA ratio greater than 1. Poly(A) RNA was transformed into labeled cRNA by the Roswell Park Cancer Institute Microarray and Genomics Core Facility (Buffalo, NY). cRNA from each pool was fragmented and its quality evaluated with Affymetrix GeneChip Test3 arrays (Santa Clara, CA) by comparing 3′:5′ signal ratios of housekeeping genes. High-quality cRNA (3′:5′ signal ratio near 1) was subsequently hybridized to Affymetrix RGU34A GeneChips, and chips were scanned with the Affymetrix 428 scanner.

### Data Analysis

Cell intensity files (.CEL) files were generated with Affymetrix Microarray Suite (MAS) 5.0 software (Affymetrix) and probe-level data were background subtracted and normalized, and gene expression was summarized using the MAS 5.0 algorithm included in the Bioconductor Affy package for R, version 1.6.1 (Ihaka and Gentleman 1996). Gene expression data from *n* = 3 GeneChips in each exposure group were averaged, and changes in gene expression were calculated as the average change versus gene expression for the *n* = 3 GeneChips from the vehicle-treated control group. Cluster analysis was performed with TIGR Microarray Experiment Viewer (Saeed et al. 2003). The gene expression profiles associated with TCDD, PeCDF, PCB126, and PCB153 exposures were assessed by principal components analysis (PCA) with the covariance value distance metric (Raychaudhuri et al. 2000) to evaluate relationships between exposure groups. Genes co-expressed during various exposure conditions were identified by Pavlidis template matching (PTM; Pavlidis and Noble 2001). For each PTM analysis, gene expression profile templates were constructed by designating relative gene expression ratios for each exposure condition. Gene expression data were filtered for genes that matched each template based on the Pearson correlation (*R* ≥ 0.9). Template matching genes were subjected to Euclidean distance hierarchical clustering. Genes were annotated with GenBank accession numbers by Affymetrix MAS 5.0 and TIGR Resourcerer gene annotation tool (Tsai et al. 2001), and official gene names were provided by the Rat Genome Database (http://rgd.mcw.edu/). Expressed sequence tags without annotation were filtered from PTM outputs, thus restricting gene sets to annotated genes. Promoters of selected genes were mapped for DREs using MatInspector Professional (Quant et al. 1995). Quantitative gene expression estimates obtained by microarray analysis were validated by two-step real-time reverse-transcriptase polymerase chain reaction (RT-PCR) for selected genes.

### Real-Time RT-PCR Validation of Gene Expression

Reverse transcriptase reactions (80 μL) contained 20 μg total RNA, 0.5 mM dNTP mix, and 15 ng/μL random primers, 1 × first-strand buffer, 10 mM dithiothreitol, 27 U Rnasin RNase inhibitor (Promega, Madison, WI), and 800 U superscript reverse transcriptase (Invitrogen). A mixture containing total RNA, dNTPs, and random primers was heated to 65°C for 5 min to denature the RNA and then immediately placed on ice. The remaining components of the reaction mixture were then added to the RNA, and cDNA synthesis was performed at 42°C for 60 min. Reactions were terminated by heating to 70°C for 10 min.

PCR primers were selected from GenBank database sequences with Primer3 software (Rozen and Skaletsky 2000). Primer sequences were between 20 and 22 bp, contained at least one 3′-GC clamp, displayed a maximal *T_m_* (melting temperature) difference of 1°C, a maximal poly-X value of 3, maximal 3′-complementarity of 2, and a *T_m_* between 60 and 62°C. Nonspecific mispriming was managed by a mispriming threshold of 10.0 in the rodent mispriming library. The primer sequences used in the present study are shown in Table 1.

Real-time PCR was performed with the SYBR Green PCR kit from Applied Biosystems (Foster City, CA) according to the manufacturer’s instructions. PCR reactions (25 μL) contained diluted cDNA, 1 × SYBR Green buffer, 3 mM MgCl_2_, 0.2 mM dNTP mix, 0.2 μM left and right primers, 10 nM fluorescein, and 1.1 U of Amplitaq Gold polymerase. The reaction was initiated by incubation at 95°C for 10 min and followed by 40 cycles of denaturation at 95°C for 15 sec and primer annealing/extension at 60°C for 1 min. Sample fluorescence was evaluated during the annealing/extension step. Upon completion of thermocycling, the specificity of each reaction was evaluated by melting analysis. Samples were heated to 95°C for 2 min and cooled to 55°C. The temperature was maintained at 55°C for 15 sec to analyze sample fluorescence, and the temperature was increased by increments of 0.5°C followed by 15 sec of fluorescence analysis for a total of 80 cycles.

The efficiency of each primer set was validated over a range of cDNA concentrations. Primer pairs that demonstrated reaction efficiencies between 85 and 103%, concentration vs. fluorescence slope factors between −1.3 and −1.7, and concentration versus fluorescence correlation coefficients between 0.98 and 1.0 were accepted for further use. After primer validation, PCR reactions were performed with a single cDNA concentration, and the threshold cycle (*C_t_*) was determined for each reaction. The difference (Δ*C_t_*) between the threshold cycle for the target gene and endogenous control gene (18S RNA) was calculated for each sample, and the 18S normalized relative expression of each gene was calculated by the comparative method according to the Applied Biosystems’ User Bulletin No. 2 for the ABI Prism Sequence Detection System (Applied Biosystems), as described by Imasato et al. (2002).

## Results

### Dosimetry and Liver Pathology from the NTP Cancer Bioassay

Tissue dosimetry and liver pathology for each 13-week HAH exposure are detailed in Table 2. The dosimetry values indicate that the AhR ligands (TCDD, PeCDF, PCB126) exhibit more pronounced hepatic accumulation, relative to administered dose, than does the non-AhR ligand PCB153. It is important to note that the exposure to PCB153 is in micrograms per kilogram per day (μg/kg/day), whereas the level of PCB153 in the liver is in units of nanograms per gram of liver (ng/g liver), supporting the greatly reduced relative hepatic accumulation of PCB153. Preferential hepatic accumulation of TCDD, PeCDF, and PCB126 is consistent with *CYP1A2* serving as a sequestering protein for AhR ligands (Van den Berg et al. 1994). There was a statistically significant increase in the incidence of hepatic hypertrophy for all exposures compared with the respective vehicle control animals. Liver hypertrophy was mild to moderate among female rats from the TCDD exposure group, minimal to mild in the PCB126 exposure group, and minimal in rats exposed to PeCDF or PCB153. A limited and non-significant number of animals exposed to TCDD for 13 weeks displayed multinucleated hepatocytes.

**Figure 1:**
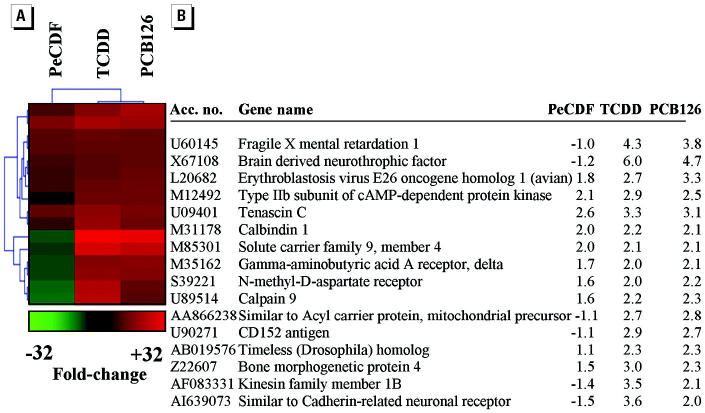
Hepatic genes differentially expressed during TCDD and PCB126 exposures but not during PeCDF exposure. Acc. no., GenBank accession number. PTM was used to identify genes co-expressed after exposure to TCDD and PCB126 but not PeCDF for 13 weeks. Matching genes conformed to a template where the relative expression ratios for toxicant versus vehicle exposure were PeCDF = 0.2, TCDD = 1, and PCB126 = 1 (*r* ^2^ = 0.9). The PTM output was further refined to include only those genes differentially expressed ≥2-fold in livers of rats exposed to TCDD and PCB126 compared with vehicle control animals. (*A*) PTM diagram showing co-expressed genes. The color key indicates the magnitude of change. (*B*) Average fold changes for *n* = 3 Affymetrix GeneChips (each chip represents pooled RNA from two animals). Accession numbers and gene names are from GenBank (http://www.ncbi.nlm.nih.gov/entrez/query.fcgi?db=nucleotide).

There was an increased incidence and severity of liver hypertrophy for the TCDD and PCB126 exposures relative to the other AhR ligand, PeCDF. PTM was employed to identify genes associated with the more pronounced hepatic hypertrophy in the TCDD and PCB126 groups relative to the PeCDF group (Figure 1). We were unable to identify genes that were selectively repressed, but we did identify a limited subset of genes selectively induced by TCDD and PCB126 compared with PeCDF. These genes were functionally classified as neurotransmitter and endocrine signaling genes.

### Effects of HAH Exposure on Global Hepatic Gene Expression

**Figure 2:**
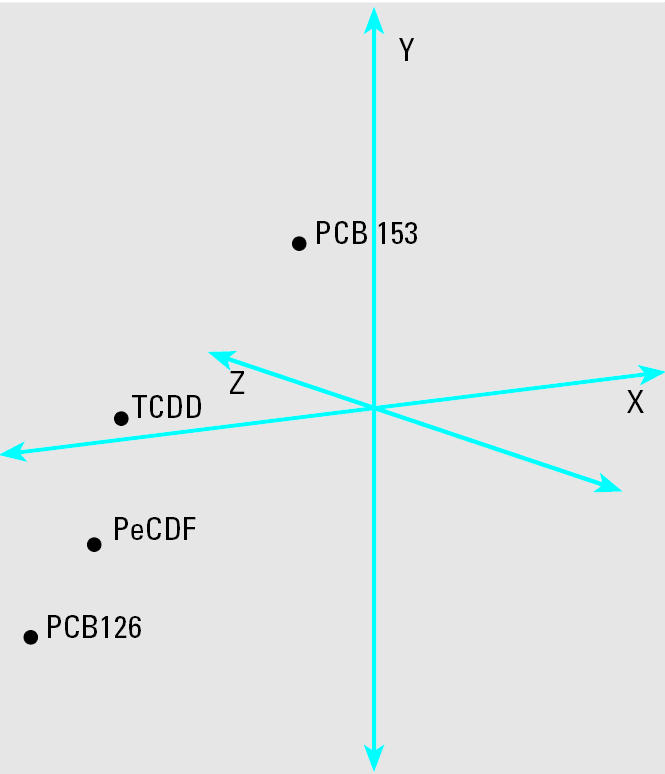
Relationship between global hepatic gene expressions resulting from TCDD, PeCDF, PCB126, or PCB153 exposures. The global hepatic gene expression profiles for rats exposed to each toxicant were analyzed by PCA. The principal components (X, Y, Z) for the gene expression of rats exposed to AhR ligands TCDD, PeCDF, and PCB126 were localized near a single PCA quadrant, whereas the principal component for the nonAhR ligand PCB153 was located in a distant quadrant.

TCDD, PeCDF, and PCB126 activate AhR, whereas PCB153 displays little or no binding affinity for AhR. PCA was employed to determine whether the relationship between global gene expression profiles for each chemical would be related to their AhR affinities (Figure 2). The principal components for TCDD, PeCDF, and PCB126 exposures were spatially co-localized along the *x*-axis of the PCA plot (PeCDF and PCB126 were localized to the same quadrant, whereas TCDD was proximally located in the adjacent quadrant). The principal component for the PCB153 exposure was unique from those of AhR ligands.

**Figure 3:**
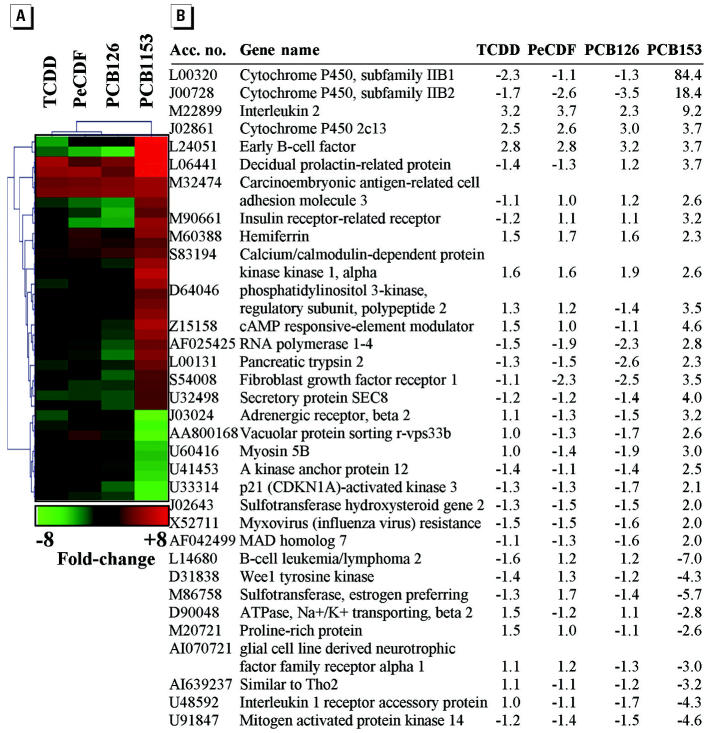
Hepatic genes differentially expressed during PCB153 exposure but not during exposure to PCDD, PeCDF, PCB126. Acc. no., GenBank accession number. PTM was used to identify differentially expressed genes in livers of rats exposed to PCB153 compared with rats exposed to AhR ligands. Matching genes conformed to a template where the relative expression ratios for toxicant versus vehicle exposure were TCDD = 0, PeCDF = 0, PCB126 = 0, and PCB153 = 1 (*r* ^2^ = 0.9). The PTM output was further refined to include only those genes differentially expressed ≥2-fold in livers of rats exposed to PCB153 compared with vehicle control animals. (*A*) PTM diagram showing genes differentially expressed during PCB153 exposure. The color key indicates the magnitude of change. (*B*) Average fold changes for *n* = 3 Affymetrix GeneChips (each chip represents pooled RNA from two animals). Accession numbers and gene names are from GenBank (http://www.ncbi.nlm.nih.gov/entrez/query.fcgi?db=nucleotide).

Because the global gene expression profile associated with PCB153 exposure differed from that of TCDD, PeCDF, and PCB126, PTM was employed to identify genes that were selectively induced or repressed by PCB153 (Figure 3). PCB153 does not bind to AhR, and genes activated or repressed during PCB153 exposure are likely regulated by an AhR-independent mechanism. Liver from PCB153-exposed rats exhibited a unique class of differentially expressed genes compared with rats exposed to AhR ligands, with *CYP2B1* being the most up-regulated by PCB153. PCB153 exposure also produced the differential expression of proinflammatory genes interleukin 2 (*IL2*) and interleukin 1 (*IL1*) and myxovirus (influenza virus) resistance (*MX1*) and apoptosis-related genes B-cell leukemia/lymphoma 2 (*BCL-2*) and Wee1 tyrasine kinase (*WEE1*).

**Figure 4:**
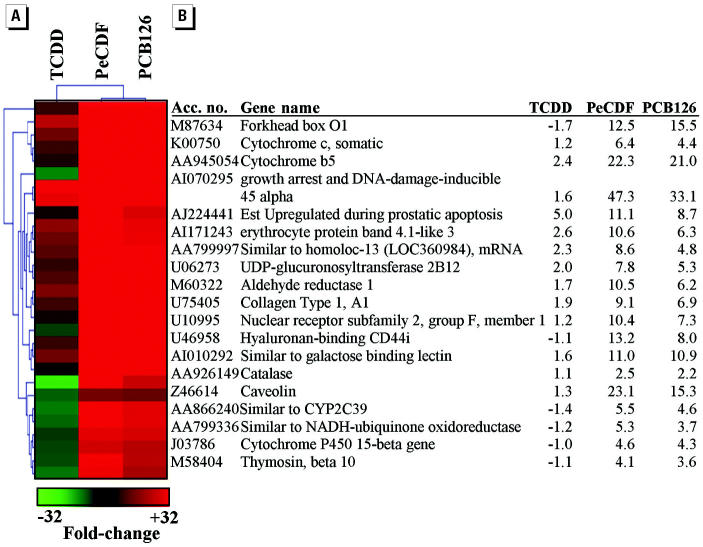
Hepatic genes activated or repressed during PeCDF and PCB126 exposure but not during TCDD exposure. PTM was used to identify genes co-expressed after exposure to PeCDF and PCB126 but not TCDD for 13 weeks. Acc. no., GenBank accession number. Matching genes conformed to a template where the relative expression ratios for toxicant versus vehicle exposure were TCDD = 0.2, PeCDF = 1, and PCB126 = 1 (*r* ^2^ = 0.9). The PTM output was further refined to include only those genes differentially expressed ≥2-fold in livers of rats exposed to PeCDF and PCB126 compared with vehicle control animals. (*A*) PTM diagram showing co-expressed genes. The color key indicates the magnitude of change. (*B*) Average fold changes for *n* = 3 Affymetrix GeneChips (each chip represents pooled RNA from two animals). Accession numbers and gene names are from GenBank (http://www.ncbi.nlm.nih.gov/entrez/query.fcgi?db=nucleotide)

Global hepatic gene expression profiles of animals exposed to PeCDF more closely resembled those of PCB126-exposed animals than of TCDD-exposed animals, indicating that PeCDF and PCB126 may co-regulate a unique group of genes that are not differentially expressed relative to TCDD exposure. This subgroup of genes may be activated by an AhR-independent mechanism unique to PeCDF and PCB126. PTM was used to identify genes that were selectively activated by PeCDF and PCB126 compared with TCDD (Figure 4). A total of 29 different genes were identified, all of which were mutually induced by PeCDF and PCB126. These genes included those coding for metabolic enzymes (cytochrome P450 15-beta gene, *CYP2C39*, NADH dehydrogenase) and oxidative stress response genes [catalase (*CAT*), cytochrome *c* oxidase (*COX*)].

**Figure 5:**
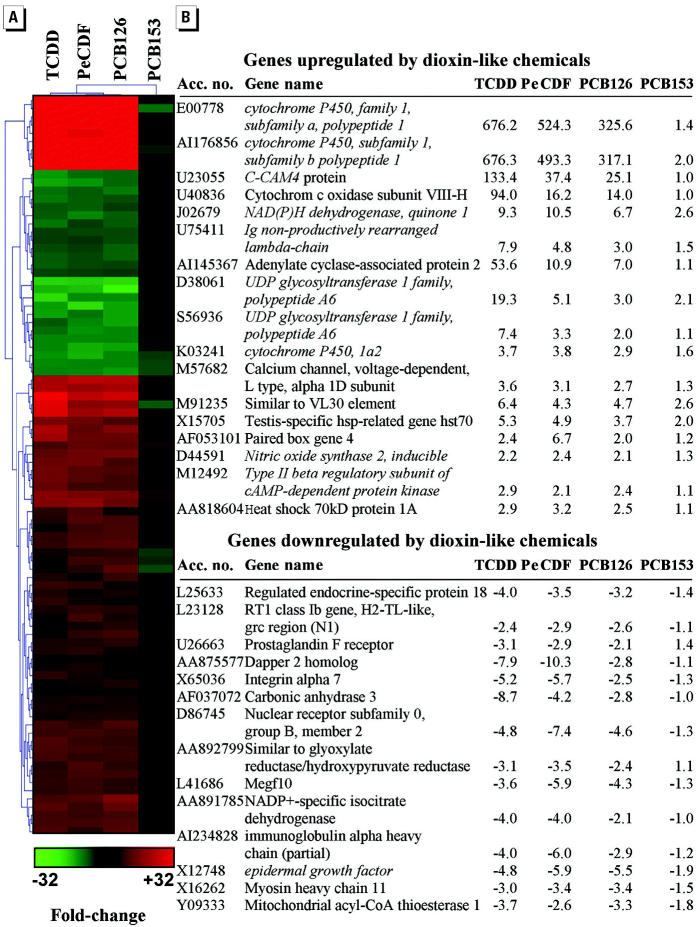
Identification of novel dioxin-responsive genes. Gene expression data were clustered by PTM to identify co-expressed genes in livers of rats exposed to AhR ligands TCDD, PeCDF, and PCB126 for 13 weeks. Acc. no., GenBank accession number. Matching genes conformed to a template where the relative expression ratios for toxicant versus vehicle exposure were TCDD = 0.8, PeCDF = 0.8, PCB126 = 0.8, and PCB153 = 0.1 (*R* ^2^ = 0.9). Each row represents a separate gene; each column specifies the toxicant treatment (*n* = 3 replicate arrays per toxicant). The PTM output was further refined to include only genes differentially expressed ≥2-fold in livers of rats exposed to PeCDF and PCB126 compared with control animals. The color key indicates the magnitude of change. Italicized genes were previously shown to respond to dioxin. Accession numbers and gene names are from GenBank (http://www.ncbi.nlm.nih.gov/entrez/query.fcgi?db=nucleotide).

PTM was also employed to identify genes co-expressed during TCDD, PeCDF, and PCB126 exposures because differential expression of these genes may be associated with AhR-mediated pathology (Figure 5). Many of the TCDD-inducible genes identified by PTM represented classic dioxin-inducible genes, including *CYP1A1*, *CYP1B1*, *CYP1A2*, NAD(P)H-menadione oxidoreductase, immunoglobulin M, and UDP glycosyltransferase 1 family, polypeptide A1. Although cytochrome *c* oxidase has been associated with TCDD induction, *COX8H* represents a novel dioxin-inducible isoform of this gene. Carcinoembryonic-cell adhesion molecule 4 (*C-CAM4*) and adenylate cyclase–associated protein 2 (*CAP2*) were also induced during TCDD, PeCDF, and PCB126 exposures. Several genes were coordinately down-regulated by TCDD, PeCDF, and PCB126 but not by PCB153, including epidermal growth factor and mitochondrial thioesterase.

*C-CAM4* and *CAP2* were highly induced by all three AhR ligands but have not been linked to AhR-mediated transcriptional regulation. The enhanced expression of *C-CAM4* and *CAP2* was verified by real-time RT-PCR (Table 3). To determine whether *C-CAM4* and *CAP2* are direct AhR target genes, the 5′-regulatory sequences of these genes were modeled by MatInspector Professional to identify potential *cis*-acting DRE sequences within these gene promoters. Neither gene promoter contained DRE consensus sequences.

The expression of several additional genes was also verified by real-time RT-PCR (Table 3). In general, there was relatively good association between the up- or down-regulation of genes according to Affymetrix GeneChip analysis and real-time RT-PCR methods. *CYP1B1*, *C-CAM4*, and *CAP2* were consistently increased in livers of rats exposed to AhR ligands (TCDD, PeCDF, PCB126), whereas livers of PCB153-exposed rats exhibited little or no change. *CYP3A9* and serine proteinase inhibitor, clade A (*SERPIN7A*) were down-regulated by exposure to AhR ligands, whereas somatostatin was down-regulated by exposure to AhR ligands and PCB153. Real-time RT-PCR also suggests that carboxylesterase 3 (*CES3*) was down-regulated by AhR ligands.

## Discussion

The TEF classification scheme has been used for many years to facilitate risk assessment for individual congeners and mixtures of dioxin-like PCDDs, PCDFs, and PCBs (Becher et al. 1998; Finley et al. 2003; Flesch-Janys et al. 1998; Van den Berg et al. 1998). However, there is some question about whether TEF values are predictive of long-term toxicologic end points, including cancer (Safe 1994). The present study evaluated hepatic gene expression during a 13-week interim sacrifice from a 2-year chronic toxicity and carcinogenicity study of TCDD, PeCDF, PCB126, and PCB153 in female Harlan SD rats. The TCDD, PeCDF, and PCB126 exposures from this study produced cholangiocarcinoma, hepatocellular adenoma, toxic hepatopathy, multinucleated hepatocytes, diffuse fatty change, and liver pigmentation after 2 years of exposure (NTP 2004a, 2004b, 2004c). Liver tumor incidence was not equivalent among AhR ligands but instead was higher in animals exposed to TCDD and PCB126 compared with animals exposed to PeCDF at toxicologically equivalent doses. Similar results were seen for the incidence and severity of hepatic hypertrophy at 13 weeks, where PeCDF produced less hypertrophy than TCDD and PCB126 (Table 2). The 13-week PeCDF exposure also induced substantially less *CYP1B1* mRNA compared with TCDD and PCB126, as shown by RT-PCR (Table 3). Thus, it is possible that enhanced AhR activation by TCDD and PCB126 may be responsible for the increased liver pathology of TCDD- and PCB126-exposed rats compared with PeCDF-exposed rats. Consistent with these findings, our laboratory previously reported more CYP1-mediated ethoxyresorufin-*O*-deethylase activity in liver microsomes from TCDD- and PCB126-exposed female SD rats compared with PeCDF-exposed rats after 13 weeks of exposure (Shubert et al. 2002). Thus, the 200 ng/kg/day PeCDF exposure is less effective in activating AhR-dependent gene expression and less toxic regarding hepatic hypertrophy and carcinogenicity compared with 100 ng/kg/day TCDD and 1,000 ng/kg/day PCB126 exposures.

An alternative possibility of the enhanced toxicity of TCDD and PCB126 exposures compared with PeCDF is that these chemicals activate a unique subgroup of genes that is not activated by PeCDF, thus enhancing toxicity. In support of this possibility, DNA microarray analysis revealed a subset of differentially expressed genes in TCDD- and PCB126-exposed rat liver that were relatively unchanged during PeCDF exposure (Figure 1). These genes were functionally related to neurotransmitter and neuroendocrine signaling. Activation of neuroendocrine signaling by TCDD has been demonstrated in other tissues, including monkey hypothalamus and rodent pituitary and adrenal (Pitt et al. 2000; Shridhar et al. 2001). Subchronic exposure to TCDD and PCB126 may therefore stimulate hepatic neuroendocrine signaling. Activation of hepatic neuroendocrine cells by these chemicals may also be a symptom or contributor to chemical-induced liver hypertrophy, which was substantially less in PeCDF-exposed rats.

AhR activation by PCDDs, PCDFs, and coplanar PCBs is a hallmark of exposure to dioxin-like chemicals and is likely implicated in their toxicity. AhR activation has also been attributed to the direct activation of some dioxin-responsive genes (Mimura and Fujii-Kuriyama 2003; Schrenk 1998; Sutter and Greenlee 1992). However, there is little knowledge regarding how many genes are activated or repressed in an AhR-dependent mechanism during sub-chronic exposure to AhR ligands. The present study used PCA to address relationships between exposure to traditional AhR ligands (TCDD, PeCDF, and PCB126) and the non-AhR ligand PCB153. PCA has been previously employed to compare genomic profiles of toxicants (Heijne et al. 2003), identify common molecular effects among potential drug candidates (Shi et al. 2000), and predict treatment prognosis (Ringberg et al. 2001). PCA revealed a unique association among hepatic gene expression profiles produced by exposure to dioxin-like toxicants, where global gene expression profiles for rats exposed to PeCDF and PCB126 were very similar and closely related to the gene expression profile for TCDD exposure (Figure 2). The global gene expression profile for exposure to the noncoplanar PCB153, however, was substantially different from that of the dioxin-like AhR ligands. Prominent differences between PCB153-mediated and AhR-ligand–mediated gene expression suggest that subchronic exposure to TCDD, PeCDF, and PCB126 has an extensive impact on global hepatic gene expression that involves many genes. Furthermore, it is important to note that the expression or repression of the genes examined may result from direct regulation by the AhR, events further downstream from a direct regulation of some gene by the AhR, and/or a response to the tissue injury resulting from the genes directly or indirectly regulated by the dioxin-like chemicals via the AhR.

PCB153 is the most prevalent PCB congener in biologic tissues (Kimbrough 1995; Krogaenas et al. 1998; Safe 1994). It is also extraordinarily persistent and its half-life may exceed 100 years in marine sediments (Jonsson et al. 2003). PCB153 exposure has been associated with various biologic effects including developmental toxicity (Kuchenhoff et al. 1999) and induction of *CYP2B1* and other phenobarbital-responsive genes (Connor et al. 1995). Although PCB153 does not bind to AhR and produced minimal hepatic hypertrophy after 13 weeks of subchronic exposure, PCB153 did promote differential expression of several biomarker genes for liver injury (Figure 3). Thus, gene expression profiling may be a more sensitive gauge of PCB153 toxicity than standard histology, and this hypothesis will be tested with low-dose PCB153 exposures in future experiments. PCB153 activates an acquired immune response in mice (Smialowicz et al. 1997). PCB153 exposure in this study was associated with differential gene expression of proinflammatory cytokines, including *IL1*, *IL2*, and the immune response gene *MX1*. PCB153 exposure decreased expression of the apoptotic genes *BCL-2* and *WEE1*. *BCL-2* and *WEE1* are responsive to p53 and are down-regulated during apoptosis (Bishay et al. 2000; Leach et al. 1998). PCB153 exposure selectively enhanced expression of cAMP response element modulator (CREM) protein. *CREM* gene activation is a signature response to liver regeneration after hepatocyte injury. *CREM* mRNA is increased after partial hepatectomy in wild-type mice and liver regeneration is inhibited in *CREM*^(−/−)^ mice (Servillo et al. 1998). The identification of these and other PCB153-responsive genes in the present study provides new targets for future mechanistic studies of PCB153 toxicity.

The mutual AhR binding affinity of TCDD, PeCDF, and PCB126 is likely responsible for strong similarity between global gene expression profiles produced by these chemicals. However, PCA also revealed that gene expression profiles associated with PCB126 and PeCDF exposures were more closely related to each other than to TCDD. On the basis of this finding, it is possible that PeCDF and PCB126 activate a unique group of genes not activated during TCDD exposure.

We identified a limited subset of genes activated by PeCDF and PCB126 but not TCDD (Figure 4). Induction of *CAT*, cytochrome *b*_5_ (*CYB5*), and *COX* oxidatative stress response genes (Poulsen et al. 2000) suggests that PeCDF and PCB126 exposures are capable of inducing oxidative stress. PeCDF and PCB126 also induced growth arrest and DNA-damage-inducible 45 (Gadd45) expression, a DNA-damage–inducible gene product (Sheikh et al. 2000). Induction of Gadd45 during PeCDF and PCB126 exposures may indicate oxidative DNA damage in liver from animals exposed to these toxicants. Oxidative stress was previously reported during AhR activation (Dalton et al. 2002). Interestingly, however, oxidative stress response genes were not activated in livers from animals exposed to TCDD in the present study. It is interesting that PeCDF produced less hypertrophy than did TCDD, yet was more effective in activating the expression of oxidative stress response genes. These data may indicate that PeCDF and PCB126 exposures promote oxidative stress through a unique, AhR-independent mechanism and/or that the responses are secondary to liver injury produced during the subchronic exposure.

AhR activation plays a critical role in many end points of TCDD toxicity [Agency for Toxic Substances and Disease Registry (ATSDR) 1998; Hahn 2002; Mimura and Fujii-Kuriyama 2003; Van den Berg et al. 1998]. PCA from the present study suggests that subchronic exposure to AhR ligands causes differential expression of numerous genes. Although a few AhR target genes have been identified in previous studies, many members of the AhR gene battery remain unknown. Genomic and proteomic approaches provide valuable opportunities to elucidate additional genes involved in AhR signal transduction and hepatotoxic responses to dioxin-like chemicals.

PTM revealed genes specifically induced or repressed by AhR ligands TCDD, PeCDF, and PCB126 but not by PCB153 (Figure 5). Many of these genes were previously classified as being dioxin responsive, which validated the efficacy of the PTM approach and further verified RNA sample integrity. PTM also revealed several genes, including *C-CAM4*, *CAP2*, *SERPIN7A*, *CES3*, and expressed sequence tags (ESTs) not yet associated with the AhR signaling pathway (Table 3).

*C-CAM4* represents a novel dioxin-responsive gene. In the present study, *C-CAM4* was selectively induced in rats exposed to AhR ligands, but not in rats exposed to PCB153, and was among the genes most highly up-regulated by TCDD exposure (Table 3, Figure 5). *C-CAM4* does not contain a promoter-based DRE sequence, which suggests that it may not be regulated directly by AhR.

C-CAM4 is a recent addition to the carcinoembryonic antigen (CEA) family of cell adhesion molecules (Earley et al. 1996). CEA immunoglobulins demonstrate important roles in growth and differentiation. Although most soluble CEA molecules are unable to mediate intercellular associations, C-CAM4 actively promotes cell adhesion (Lin et al. 1998). C-CAM1 is a secretory paralog of C-CAM4, is expressed during hepatocyte differentiation (Cheung et al. 1993; Thompson et al. 1993), and is selectively down-regulated in hepatocellular carcinoma (Cheung et al. 1993; Thompson et al. 1993). TCDD has been associated with hepatocellular carcinoma in female Spartan SD rats (Kociba et al. 1978) and with hepatocellular adenoma and cholangiocarcinoma in a recent NTP study of female Harlan SD rats (NTP 2004a). The NTP study also found evidence of other hepatotoxic responses after 2 years of chronic exposure to TCDD, including hepatocyte hypertrophy, multinucleated hepatocyte, diffuse fatty change, bile duct hyperplasia, bile duct cyst, oval cell hyperplasia, necrosis, pigmentation, inflammation, nodular hyperplasia, portal fibrosis, cholangiofibrosis, and toxic hepatopathy (NTP 2004a). No major evidence for hepatotoxicity was observed after 13 weeks of exposure to TCDD, but livers from female rats exposed for this time period were hypertrophic. The increased expression of secreted *C-CAM4* in these rats may suggests that TCDD initiates a cellular transition from membrane-bound *C-CAM1* expression in normal tissue to secreted *C-CAM4* expression during hypertrophy and neoplastic transformation. Although *C-CAM4* may be a potential marker for disrupted cell differentiation in livers of animals exposed to dioxin-like toxicants, it will be important in future studies to investigate protein expression in conjunction with gene expression

Like *C-CAM4*, *CAP2* is a novel dioxin-responsive gene that exhibits dynamic activation in the presence of AhR ligands (Table 3, Figure 5). The *CAP2* promoter is also devoid of XRE sequences. *CAP2* mRNA is expressed at moderate to low levels in normal rat liver tissue (Swiston et al. 1995) but is markedly induced by TCDD and related compounds. It is unclear whether *CAP2* activity is implicated in the preneoplastic hepatotoxic effects of dioxin in rats, but a yeast homolog of *CAP2* associates with the actin cytoskeleton (Lila and Drubin 1997), is responsible for the posttranslational posttranslational processing of Ras, and serves as an effector for Ras-dependent activation of adenylyl cyclase (Shima et al. 1997). Ras expression was increased in altered hepatic foci from a diethyl nitrosamine/TCDD tumor initiation and promotion study (Sills et al. 1994). A Ras-related mechanism suppressed *CYP1A1* expression, potentially serving as a negative feedback mechanism that rectifies *CYP1A1* levels in the presence of TCDD (Reiners et al. 1997). Although the AhR pathway may indeed have cross talk with the adenylyl cyclase pathway, the specific details of these interactions are unclear. *CAP2* may play an important role in this signaling cross talk and warrants further characterization. As with *C-CAM4*, future studies will investigate protein expression in conjunction with the expression of this gene.

This study represents one of the first attempts to characterize hepatic gene expression in the context of subchronic exposure to carcinogenic doses of dioxin-like chemicals. DNA microarrays and/or RT-PCR successfully identified novel dioxin-responsive genes that were either up-or down-regulated after exposure to AhR ligands (TCDD, PeCDF, PCB126) but not after exposure to the non-AhR ligand PCB153. Future studies are needed to assess the species- and tissue-specific expression of these genes and their respective functional proteins to establish whether these differentially expressed genes may be a response to and/or lead to the carcinogenic and/or noncarcinogenic effects of these compounds in humans and laboratory animals. Together, these findings may help to elucidate some of the fundamental features of dioxin toxicity and may further clarify the biologic role of the enigmatic AhR signaling pathway.

## Figures and Tables

**Table 1:** Primer sequences for real-time RT-PCR.

GenBank accession no.*^a^*	Gene	Primer sequences (5′ to 3′)
U09540	*CYP1B1*	CGTCTGATGCTTTCAGCAAAGG
		GCAGGCTTTCCAACTAAGCCAG
U23055	*C-CAM4*	CTCGTCTCCTCAGAGGGCAGATTC
		ACAGCGTCTACGGTGACTTGGG
Al145367	*CAP2*	ATCACCGTCGATAACTGCAAG
		CCCATTACCTGGATCTGAATG
U46118	CYP3A9	CCACCAGCATGAAAGACATC
		GTCCTGTGGGTTGTTAAGGG
M63991	*SERPIN7A*	TCTGGCTCTAGCACCCAAAC
		GATCAAATGCTGGAAGCCC
M25890	*SST*	CAGAACTGCTGTCTGAGCCC
		AGCTCCAGCCTCATCTCGTC
X65296	*CES3*	GGCCATTTCTGAGAGTGGTGTG
		GCAGGCAATGAACCATAACAGC
V01270	*Ribosomal 18S RNA*	GAGCGAAAGCATTTGCCAAG
		GGCATCGTTTATGGTCGGAA

a From GenBank (http://www.ncbi.nlm.nih.gov/entrez/query.fcgi?db_nucleotide)

**Table 2:** Summary of the 13-week dosimetry and liver pathology data from the NTP cancer bioassay.*^a^*

	TCDD		PeCDF		PCB126		PCB153	
	Control	100 ng/kg/day	Control	200 ng/kg/day	Control	1,000 ng/kg/day	Control	1,000 μg/kg/day
Liver levels (ng/g)	BLOQ	18.3 ± 0.8	BLOQ	132.9 ± 47.4	BLOQ	412.6 ± 73.5	BLOQ	9,250 ± 2,061
Liver hypertrophy	1 (1.0)	10* (2.3)	0	7* (1.0)	0	10* (1.7)	0	9* (1.2)
Multinucleated hepatocytes	0	3 (1.0)	0	0	0	0	0	0
Diffuse fatty change	0	2 (1.0)	0	2 (1.0)	0	0	0	0
Pigmentation	0	0	0	2 (1.0)	0	0	0	0

Abbreviations: BLOQ, below level of quantification; N/A, not applicable.

a Dosimetry values are the mean ±SD of 10 rats per group. Values for each pathologic end point represent the incidence or number of rats (of 10) that exhibit the response. The mean severity score is given in parentheses (1, minimal; 2, mild; 3, moderate; 4, marked).

* Statistically significant (*p* < 0.05) increase in the incidence of a given pathologic response relative to the respective control group.

**Table 3:** Differential gene expression in liver from rats exposed subchronically (13 weeks) to HAHs.

	TCDD	PeCDF	PCB126	PCB153
*CYP1B1*				
RT-PCR	256 (236.3 to 277.3)	59.7 (48.3 to 73.8)	106.4 (98.2 to 115.3)	−1.2 (−1.6 to 1)
Microarray	676.3 (470.8 to 881.8)	493.3 (310.1 to 676.5)	317.1 (41.1 to 593.1)	2.1 (1 to 3.2)
*C-CAM4*				
RT-PCR	4.1 (3.8 to 4.4)	2.5 (2.3 to 2.7)	3.5 (2.6 to 4.7)	−1.1 (−1.3 to 1)
Microarray	133.4 (112.1 to 154.7)	37.4 (25.5 to 49.3)	25.1 (4 to 46.2)	1.1 (−0.7 to 1.5)
*CAP2*				
RT-PCR	168.9 (147.9 to 192.9)	20.2 (17.8 to 22.9)	119.4 (104 to 137.2)	−1.3 (−1.5 to −1.1)
Microarray	53.6 (31.1 to 76.1)	10.9 (4 to 17.8)	7.6 (2 to 13.2)	1.1 (−0.8 to 1.4)
*CYP3A9*				
RT-PCR	−1024 (−1201.8 to −872.5)	−4.2 (−5 to −3.5)	−28.5 (−30.2 to −26.9)	−1.8 (−2.1 to −1.5)
Microarray	−35.4 (−53.4 to −17.4)	−2.3 (−3.3 to −1.3)	−4.5 (−5.8 to −2.2)	1.6 (0.8 to 2.4)
*SERPIN7A*				
RT-PCR	−26.6 (−30.2 to −23.4)	−29.9 (−32 to −27.9)	−8.4 (−12 to −5.9)	−1.2 (−1.5 to 1)
Microarray	−11.5 (−17.2 to −5.8)	−2.7 (−3.6 to −1.8)	−4 (−4.9 to −3.1)	1.6 (1.3 to 1.9)
*SST*				
RT-PCR	−36.8 (−46.4 to −29.1)	−1.6 (−2 to −1.2)	−1.5 (−1.6 to −1.4)	−3 (−3.5 to −2.5)
Microarray	−8 (−10.5 to −5.5)	−8.3 (−11.1 to −5.5)	−6.4 (−9.4 to −3.4)	−9.5 (−10.1 to −8.9)
*CES3*				
RT-PCR	−4.8 (−5.2 to −4.4)	−2.8 (−2.5 to −3.1)	−1.4 (−1.5 to −1.3)	2.4 (2.1 to 2.6)
Microarray	−8.1 (−9.9 to −6.3)	−2.8 (−3.8 to −1.8)	−4 (−4.4 to −3.6)	1.3 (1 to 1.6)

The average changes in hepatic mRNA for TCDD, PeCDF, PCB126, or PCB153 exposures compared with vehicle exposure were determined by real-time RT-PCR to validate microarray results. Each value represents the average (± 1 standard deviation) of three independent RNA pools (RT-PCR) or three independent GeneChips (microarray). Real-time RT-PCR values were normalized to 18S ribosomal RNA as described in “Materials and Methods.”
